# Accuracy of Cup Alignment in Total Hip Arthroplasty: A Comparison Between Portable Navigation and Goniometer

**DOI:** 10.7759/cureus.74176

**Published:** 2024-11-21

**Authors:** Tomonori Tetsunaga, Tomoko Tetsunaga, Kazuki Yamada, Takashi Koura, Tomohiro Inoue, Ryuichiro Okuda, Yasutaka Masada, Sho Muguruma, Yuki Okazaki, Toshifumi Ozaki

**Affiliations:** 1 Department of Musculoskeletal Health Promotion, Faculty of Medicine, Dentistry and Pharmaceutical Sciences, Okayama University, Okayama, JPN; 2 Department of Orthopaedic Surgery, Okayama University Hospital, Okayama, JPN; 3 Department of Medical Materials for Musculoskeletal Reconstruction, Faculty of Medicine, Dentistry and Pharmaceutical Sciences, Okayama University, Okayama, JPN; 4 Department of Orthopaedics, Okayama Medical Center, Okayama, JPN; 5 Department of Orthopaedics, Faculty of Medicine, Dentistry and Pharmaceutical Sciences, Okayama University, Okayama, JPN; 6 Department of Orthopaedic Surgery, Faculty of Medicine, Dentistry and Pharmaceutical Sciences, Okayama University, Okayama, JPN

**Keywords:** hip, navigation system, portable navigation, retrospective study, total hip arthroplasty (tha)

## Abstract

Background

Navigation systems, including portable navigation systems, used for total hip arthroplasty (THA) are useful for achieving higher cup alignment accuracy. NAVBIT, a newly available portable navigation system, uses a unique registration method, the table tilt registration. However, its accuracy is unclear. This retrospective study aimed to investigate whether THA with a portable navigation system in the lateral position with the flip technique is more accurate than THA with a cup goniometer in the supine or lateral positions.

Methodology

This study included 96 consecutive patients (77 women, 19 men) who underwent primary cementless THA using either a portable navigation system in the lateral position with the flip technique or a cup goniometer in the supine or lateral positions. The average age of the patients was 66.8 years (range = 29-91) and the average body mass index was 24.6 kg/m^2^ (range = 17.5-39.9). The accuracy of cup orientation was compared among the three groups.

Results

The absolute values of the difference in cup inclination and anteversion with the NAVBIT (2.1 ± 1.7°, 2.0 ± 1.4°) were smaller than that with the cup goniometer in the supine (3.4 ± 2.4°, 3.4 ± 2.2°) and lateral decubitus positions (3.4 ± 2.5°, 5.0 ± 3.5°). Overall, 91%, 64.5%, and 56.3% were within 5° of the target angles in the navigation, supine goniometer, and lateral goniometer groups, respectively.

Conclusions

The accuracy of cup alignment with the portable navigation system using the flip technique was significantly higher than that with the cup goniometer in the supine and lateral decubitus positions.

## Introduction

Total hip arthroplasty (THA) has been performed widely for relieving severe pain in patients with hip disorders and thereby improving their quality of life. Although surgical techniques have evolved, improving implant placement accuracy and maximizing patient benefits remain challenging. Poor cup alignment results in postoperative impingement [1], dislocation [2], accelerated polyethylene wear [3], liner damage, and limited range of motion [4]. Therefore, accurate real-time implant placement during surgery is required. Recently, computer-assisted surgery has played an important role in surgical planning. Robot-assisted surgery and CT-based navigation systems have dramatically improved the accuracy of implant placement [5,6] and can help achieve correct cup orientation, regardless of the operator’s experience [7]. Unlike conventional expensive and high-performance CT-based navigation systems, portable navigation systems are epoch-making systems that do not require preoperative CT and allow simple registration during surgery [8-10]. However, conventional portable navigation systems have two limitations. First, accurate registration is difficult in patients with obesity and pelvic deformities. Many portable navigation systems construct a pelvic reference plane by recognizing both anterior superior iliac spines (ASISs) and use it as an index for cup alignment; however, this is difficult in obese patients and those with pelvic deformities. Second, cup placement accuracy is high in the supine position. However, in the lateral position, it is difficult to create an accurate pelvic reference plane, resulting in poor accuracy [11,12]. To solve these problems, in this study, we developed a new registration method that does not require touching the ASISs and adopted a flip technique in which registration is performed in the supine position and followed by the lateral decubitus position.

This study aimed to investigate the accuracy of acetabular cup placement in THA using a portable navigation system with a flip technique. We aimed to contribute to the improvement of surgical techniques and the optimization of patient outcomes by clarifying the effects of portable navigation systems on surgical accuracy and patient rehabilitation. The results of this study are expected to advance the development of surgeons, patients, and medical technology.

This article was previously published as a preprint on Research Square [13].

## Materials and methods

Patient background characteristics

The study was approved by the University’s Health Sciences Institutional Review Board (approval number: 2310-010). All procedures were performed following the ethical standards of the institutional and/or national research committee and the 1964 Declaration of Helsinki and its later amendments or comparable ethical standards. This was a retrospective review of 96 hips of 96 consecutive patients (77 hips in 77 women and 19 hips in 19 men) who underwent primary cementless THA between April 2023 and December 2023 (Table 1). Patients who were suffering from either osteoarthritis of the hip, osteonecrosis of the femoral head, rheumatoid arthritis, or posttraumatic osteoarthritis were included. The exclusion criteria included the lack of complete routine radiological data (five hips in five patients) and revision THA (five hips in five patients). The preoperative diagnoses were osteoarthritis in 76 hips, osteonecrosis of the femoral head in 15, rheumatoid arthritis in three, and posttraumatic osteoarthritis in two. The mean age of the patients at the time of surgery was 66.8 years (range = 29-91 years), the mean height was 155.5 cm (range = 139.8-179 cm), and the mean body mass index (BMI) was 24.6 kg/m^2^ (range = 17.5-39.2 kg/m^2^). In total, 33 patients underwent THA using a portable navigation system (NAVBIT; Smith & Nephew plc, Tokyo, Japan) in the lateral decubitus position (navigation group), 31 patients underwent THA using a goniometer in the supine position (supine group), and 32 patients underwent THA using a goniometer in the lateral decubitus position (lateral group).

**Table 1:** Demographic characteristics of patients. ^a^: Data are expressed as the mean ± standard deviation (range). ^b^: Data are expressed as the number of patients. BMI: body mass index; CE angle: center edge angle

Parameters	n = 96
Age (years)^a^	66.8 ± 11.1 (29–91)
Sex: women/men^b^	77/19
Treated side: right/left^b^	58/38
Diagnosis^b^	
Osteoarthritis	76
Osteonecrosis	15
Rheumatoid arthritis	3
Posttraumatic osteoarthritis	2
Height (m)^ a^	1.55 ± 0.1 (1.39–1.79)
Weight (kg)^ a^	59.4 ± 12.8 (39.8–97.3)
BMI (kg/m^2^)^ a^	24.6 ± 4.3 (17.5–39.9)
Sharp angle (˚)^ a^	41.4 ± 4.4 (33–49)
CE angle (˚)^ a^	25 ± 12.5 (3–53)
Surgical approach^b^	
Modified Watson-Jones	96

Devices and surgical procedure

**Figure 1:**
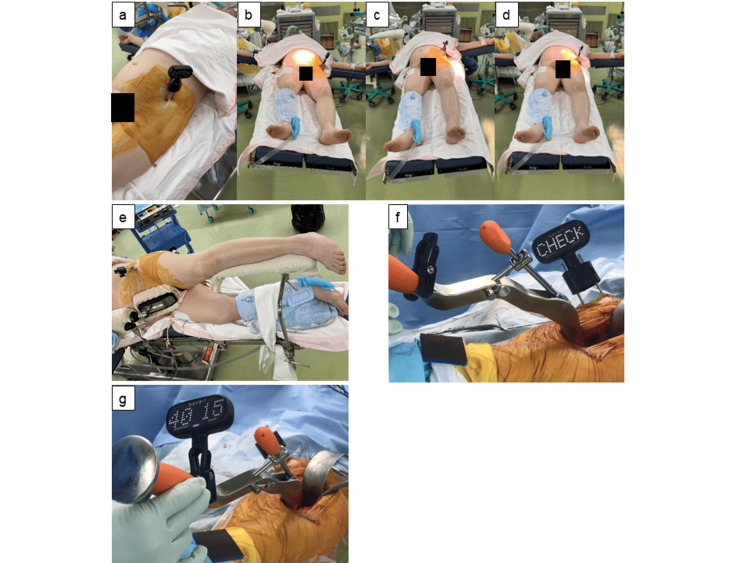
NAVBIT, a newly available portable navigation system. Insert two navigation pins parallel to the iliac crest and attach the NAVBIT sensor (a). Table tilt registration. Tilt the bed 10° to the left and register (b), tilt the bed 10° to the right and register (c), and return the bed to the horizontal position and register (d). After registration in the supine position, change the patient’s position to the lateral position and perform surgery in the lateral position (the flip technique) (e). Press fit the cup with a radiographic inclination of 40° and radiographic anteversion of 15° (f, g).

Surgery was performed using the Modified Watson-Jones approach by three operators who had performed ≥1,000 THA procedures. The navigation group used a portable navigation system (NAVBIT; Smith & Nephew plc, Tokyo, Japan; Figure 1). The NAVBIT is an inertial navigation system for THA that contains rate gyroscopes and accelerometers to generate real-time information regarding cup inclination and anteversion.

Preoperatively, 3.2-mm pins were inserted into the iliac crest on the affected side in parallel using a guide with the guide marker in the supine position. The pelvic bases of the portable navigation system were fixed with 3.2-mm pins (Figure 1a). The sensor was attached and registration was performed using the table tilt method with NAVBIT (Figures 1b-1d). The body axis was aligned with the horizontal axis for the table registration. The line connecting both the ASISs was horizontal to the ground. The gravitational vector representing the functional anteroposterior axis was obtained. Subsequently, the operating table was tilted left and right by 10°, and each gravity vector was acquired. The vector perpendicular to these two gravitational vectors was the axis of rotation, which represented the functional longitudinal axis of the patient. This functional longitudinal axis creates a functional coordinate system for calculating cup inclination and anteversion. After table registration, the surgeon placed the patient in the lateral decubitus position while maintaining sterility of the pelvic base inserted into the iliac crest by placing a sterile cup over the pelvic base (flip technique; Figure 1e).

After reaming the acetabular bone, a cementless hemisphere cup was placed on the reamed acetabulum according to the alignment determined using portable navigation or a goniometer. The radiographic inclination and anteversion angles were set to 40° and 15° for cup alignment, respectively. After the acetabular component placement, cup inclination and anteversion were measured (Figures 1f, 1g). Radiographically defined angles were displayed on a portable navigation system [14]. The cementless cup was fixed using two or three screws. A cementless stem was used on the femoral side. A trial was performed to confirm stability, leg length, and joint range of motion. A cementless stem of the same size as that used in the trial was inserted and a ceramic femoral head of the appropriate size was selected.

Postoperative management and evaluations

The postoperative protocols were the same for all patients, with recommendations for full weight-bearing as tolerated starting the day after surgery. Postoperative CT was performed according to the previously reported protocol [15]. Postoperative CT data were imported into three-dimensional templating software (ZedHip; LEXI, Tokyo, Japan). First, the pelvic coordinate system was set in the functional pelvic plane (FPP) in the coronal, sagittal, and horizontal planes. During the postoperative evaluation, the FPP, which is a horizontal plane containing both ASISs, was used as the reference plane. The radiographic cup inclination angle was measured on a slice in which the diameter of the acetabular component was maximal in the coronal plane [14]. The anatomical anteversion angle was measured similarly in the horizontal plane. The anatomical anteversion angles were then recalculated to provide the radiographic cup anteversion angles. All measurements were repeated thrice by an orthopedic surgeon, and the mean values were calculated. All the angles of the acetabular components were given as radiographically defined angles [14]. The accuracy of the acetabular component orientation was defined as the absolute value of the difference between the intraoperative record and the postoperative measurements on CT [16].

The primary endpoint was to compare the accuracy of cup orientation (the absolute value of the difference between the intraoperative record and postoperative measurement) among the three groups using postoperative CT. The secondary endpoints were intraoperative and postoperative complications. Postoperative complications were assessed three months after surgery. Loosening of the reference antenna was defined as a wobble of 2 mm or more in the screws. The proportion of patients within the safe zone (i.e. 40° ± 10° inclination and 15° ± 10° anteversion) was also assessed [17].

Sample size and statistical analysis

In a pilot study, the mean absolute values of the differences between postoperative measurements and intraoperative records for cup inclination were 2.0° with the portable navigation system and 3.5° with the goniometer in the supine position, with a standard deviation of 2.0°. Based on the effect size, in this pilot study, a power calculation (p < 0.05; power = 0.8) suggested that 29 patients were required for the trial. Variables with normal distributions were compared using one-way repeated-measures analysis of variance (ANOVA) with Tukey’s post hoc test, and variables with non-normal distributions were compared using the Kruskal-Wallis test. Values are shown as the mean ± standard deviation. P-values <0.05 were considered statistically significant. The number of outliers among the absolute values of the differences (an absolute value of the difference ≥10°) in each group was also estimated. Statistical analyses were performed using SPSS for Windows version 25 (IBM Corporation, Armonk, NY, USA).

## Results

Demographic data

Patient background characteristics are presented in Table 2. No significant differences were identified in the population data (age, sex, treated side, disease, BMI, or radiographic indices) among the three groups.

**Table 2:** Patient demographic data by groups. Data are expressed as the mean ± standard deviation and range. ^a^: One-way repeated-measures analysis of variance (ANOVA). ^b^: Kruskal-Wallis test. BMI: body mass index

	Navigation group (n = 33)	Supine group (n = 31)	Lateral group (n = 32)	P-value
Age (years)	67 ± 14.2 (29–91)	64.8 ± 11.5 (45–85)	68.4 ± 5.5 (58–81)	0.4340^a^
Sex, women/men	27/6	22/9	28/4	0.2474^b^
Treated side, right/left	19/14	21/10	18/14	0.5947^b^
Diagnosis				0.6132^b^
Osteoarthritis	25	24	27	
Osteonecrosis	5	6	4	
Rheumatoid arthritis	1	1	1	
Posttraumatic osteoarthritis	2	0	0	
Height (m)	1.56 ± 0.1 (1.40–1.79)	1.56 ± 0.1 (1.39–1.78)	1.54 ± 0.06 (1.39–1.65)	0.4800^a^
Weight (kg)	60 ± 15.4 (39.8–97.3)	59.5 ± 12.0 (48.8–93.4)	58.7 ± 10.6 (41.1–91.1)	0.9176^a^
BMI (kg/m^2^)	24.5 ± 4.8 (17.5–39.2)	24.2 ± 3.2 (19–31.6)	24.9 ± 10.6 (19–39.9)	0.8354^a^
Surgical approach				
Modified Watson-Jones	33	31	32	1^b^
Surgical position	Lateral	Supine	Lateral	
Portable navigation	○	-	-	
Alignment guide	-	〇	〇	
Bleeding (mL)	197 ± 123 (30–490)	232 ± 143 (40–950)	232 ± 138 (50–550)	0.5273^a^
Surgical time (minutes)	83.2 ± 15.5 (60–119)	94.2 ± 21.9 (70–147)	89.7 ± 20.3 (55–135)	0.1098^a^

Accuracy of cup orientation and complications

Portable navigation systems worked normally in all cases in the navigation group. The cup radiographic inclination on postoperative CT was 39.9° ± 2.9° in the navigation group, 41.1° ± 4.3° in the supine group, and 37.4° ± 3.4° in the lateral group (Table 3). Significant differences were observed between the three groups (p = 0.001). The cup radiographic anteversion on postoperative CT was 14.1° ± 5.5° in the navigation group, 16.1° ± 4.1° in the supine group, and 16.6° ± 5.9° in the lateral group, with no significant difference among the three groups (p = 0.1357).

**Table 3:** Measurements of cup angle. Data are expressed as mean ± standard deviation and range. ^a^: One-way repeated-measures analysis of variance (ANOVA). * indicates statistical significance.

		Navigation group (n = 33)	Supine group (n = 31)	Lateral group (n = 32)	P-value
Postoperative	Inclination (˚)	39.9 ± 2.9 (34.9–47.9)	41.1 ± 4.3 (31.9–48.8)	37.4 ± 3.4 (30–45)	0.001^a^*
	Anteversion (˚)	14.1 ± 5.5 (9.3–21)	16.1 ± 4.1 (8.6–22.1)	16.6 ± 5.9 (5.0–29)	0.1357^a^
Absolute values of the differences	Inclination (˚)	2.1 ± 1.7 (0–7)	3.4 ± 2.4 (0.1–8.3)	3.4 ± 2.5 (0–10)	0.0208^a^*
	Anteversion (˚)	2.0 ± 1.4 (0–5.4)	3.4 ± 2.2 (0.3–8.5)	5.0 ± 3.5 (0.5–14)	0.001^a^*

The absolute value of the difference from the values measured on postoperative CT was 2.1 ± 1.7° (range = 0°-7°) for radiographic inclination and 2.0 ± 1.4° (range = 0°-5.4°) for radiographic anteversion for the navigation group, 3.4 ± 2.4° (range = 0.1°-8.3°) for radiographic inclination and 3.4 ± 2.2° (range = 0.3°-8.5°) for radiographic anteversion for the supine group, and 3.4 ± 2.5° (range = 0°-10°) for radiographic inclination and 5.0 ± 3.5° (range = 0.5°-14°) for radiographic anteversion for the lateral group. Significant differences were observed between the three groups in both radiographic inclination and anteversion (p = 0.0208 and 0.001, respectively). There was a significantly smaller mean absolute difference in postoperative CT for the navigation group than for the supine and lateral groups for radiographic inclination (p = 0.0450 and 0.0402, respectively) and radiographic anteversion (p = 0.0453 and 0.0268, respectively).

**Figure 2:**
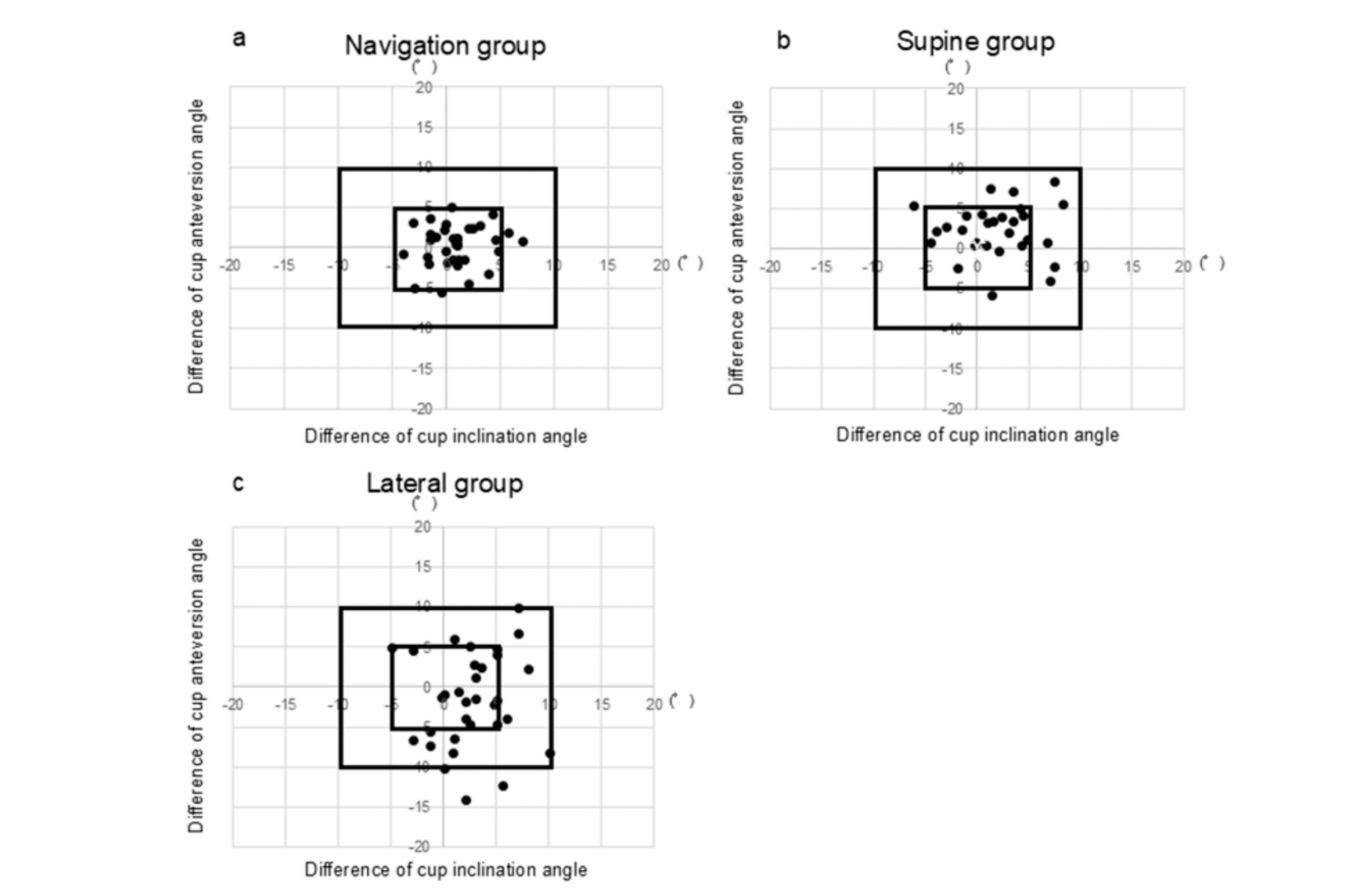
Scatter plot of the radiographic inclination and anteversion difference (postoperative CT-navigation record). a: Navigation group. b: Supine group. c: Lateral group.

All patients in the navigation and supine groups were within 10° of the target (Figures 2a, 2b). In total, 25 (84.4%) hips in the lateral group had a mean absolute error of <10° from postoperative CT measurements (Figure 2c). Overall, 30 (90.9%) patients in the navigation group, 20 (64.5%) patients in the supine group, and 18 (56.3%) patients in the lateral group were within 5° of the CT measurements (Figure 2). None of the patients showed fracture, postoperative dislocation, or required repeated surgeries for other reasons. No loosening of the reference antenna with portable navigation was observed in this series.

## Discussion

This study compared the accuracy of cup orientation between surgeries using a portable navigation system with the flip technique and a cup goniometer. The results confirmed that the accuracies of acetabular cup inclination and anteversion using a portable navigation system with a flip technique were better than those obtained using a goniometer in the supine and lateral decubitus positions.

The acetabular component orientation influences joint stability in THA [18]. Recently, various portable navigation systems have been developed for this purpose. The mean absolute values of the navigation error using NAVISWISS were 2.8° for both inclination and anteversion in the supine position [8]. Three-dimensional (3D) mini-optical navigation systems (Intellijoint HIP, Intellijoint Surgical, Inc.) and accelerometer-based portable navigation systems (HipAlign, Zimmer-Biomet) also provide clinically comparable precision and accuracy for determining the orientation of cup placement in the supine position [9]. The NAVBIT, the portable navigation system used in this study, has several advantages. First, there is no need to register the pelvis with a pointer during surgery. Second, the portable navigation system can use a flipping technique. Third, the angle displayed meets the radiographic definition without preoperative CT data.

Generally, significant differences in pelvic tilt and rotation are observed intraoperatively with the patient in the lateral decubitus position [19]. In THA performed in the lateral decubitus position, various degrees of pelvic anteroposterior tilt have been reported compared to that in the supine position [20]. Even with the use of a rigid patient fixator, clinically problematic acetabular component malposition can occur in the lateral decubitus position, especially in cup anteversion [21,22]. NAVBIT allows registration in both the supine and lateral decubitus positions. In the lateral registration, the body axis is calculated by rotating the surgical bed back and forth by 10°. However, if the pelvis is misaligned in the lateral decubitus position before registration, accurate FPP and body axis cannot be set. To solve this problem, it is necessary to perform radiographic control using fluoroscopy or radiography after fixing the body position, which is difficult for the operator. Therefore, in this study, we selected supine registration for the navigation group. In the lateral group, the pelvic tilt in the coronal, sagittal, and axial planes was corrected using radiography before surgery. However, the accuracy of pelvic alignment under radiographic control is imperfect, and pelvic alignment may change during surgery. Kanazawa et al. reported that further pelvic movement of approximately 3° in three planes was observed ranging from -11° to 20° in cup placement [20]. The navigation error of cup anteversion registered in the lateral decubitus position was significantly greater than that registered in the supine position when using an accelerometer-based navigation system (HipAlign, Zimmer-Biomet) [11,15]. This is because considerable intraoperative discrepancies in sagittal pelvic tilt result in variability in the cup anteversion angle [15].

In this study, we used the flip technique by changing the patient’s position from supine to lateral decubitus after registration. With this technique, the surgeon does not need to worry about pelvic rotation or tilt during surgery because the FPP set in the supine position is memorized even in the lateral decubitus position. Therefore, the angle displayed is not easily affected by changes in pelvic tilt or rotation. In an augmented reality (AR)-based portable navigation system and NAVISWISS, the flipping technique after registration is possible in the same manner as in NAVBIT [10]. Tsukada et al. reported that an AR-based portable navigation system using the flip technique provides more precise acetabular cup placement than an accelerometer-based portable navigation system in the lateral decubitus position [10]. Ogawa et al. reported that the absolute differences between the targeted and measured placement angles were 1.9° for inclination and 2.8° for anteversion in the lateral decubitus position [23].

Although most patients included in the present study were comparatively thin, with a mean BMI of 24.6 kg/m^2^, the presence or absence of obesity is a risk factor for acetabular component placement accuracy when THA is performed manually [24]. BMI affects the cup inclination when THA is performed with an anterolateral approach in the supine position [25]. However, CT-based navigation systems enable accurate implant placement, even in obese patients. However, many portable navigation devices register by palpating the bilateral ASISs. The digitization error of 1 cm at the ipsilateral ASIS, contralateral ASIS, and the center of the two pubic tubercles resulted in 1.8°, 4.4°, and -6.8° in anteversion, respectively [26]. It is difficult to palpate the ASISs in obese patients. Deviation of palpation in the craniocaudal direction leads to errors in inclination, and registration of ASISs over the skin leads to errors in anteversion owing to soft tissue thickness. The accuracy of portable navigation systems may decrease in obese patients. Cup malalignment (absolute value of the difference in inclination or anteversion >5°) was significantly associated with BMI in accelerometer-based portable navigation [9]. In contrast, the table tilt registration method in NAVBIT only involves turning the bed left and right in the supine position; therefore, the presence or absence of obesity does not affect this system. We believe that this is the reason why the NAVBIT used in this study had an absolute value error of 2.1° for inclination and 2.0° for anteversion, which is equivalent to or better than those in other reports.

**Figure 3:**
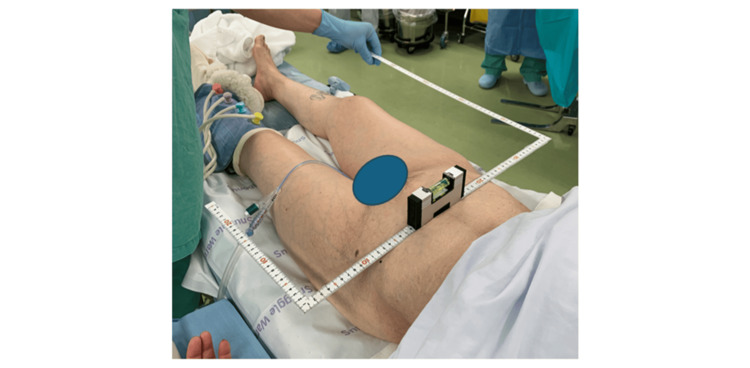
The steel square ruler and the level guide.

The present study has some limitations. First, in this study, the FPP was used rather than the anterior pelvic plane (APP) to compare accuracy among the three surgical procedures. Grammatopoulos et al. reported that with the patient in the supine position, the intraoperative anterior pelvic tilt was 1.4°, with a mean internal rotation of 1.4° and a mean adduction of 0.9° [19]. A difference in pelvic tilt makes it impossible to accurately compare cup alignment during surgery and on postoperative CT images by using the FPP as the standard plane. Therefore, the APP may be better than the FPP when comparing cup alignments. Second, this study included patients with mild deformities. Yamada et al. reported that severe pelvic deformities reduce the accuracy of navigation systems [27]. Different results may have been obtained if this study had been performed in patients with more severe deformities. However, Ueoka et al. reported that the accuracy of the navigation system was comparable between Crowe types I and IV [5]. We believe that THA in cases of mild deformity, combined with a portable navigation system, can provide the same accuracy as THA in cases of severe deformity. However, there is concern about registration accuracy in cases of pelvic obliquity due to severe degenerative scoliosis because the body axis becomes inaccurate, which affects cup inclination. In patients with hip flexion contracture or who are extremely thin, care must be taken to ensure that the pelvis is not rotated. In this system, if the pelvis is rotated during registration, an error occurs in the cup anteversion. We usually use the steel square ruler and the level guide because registration with the correct pelvic alignment is important for the use of this portable navigation system (Figure 3). Finally, the number of cases in this study was small to determine the complication rates. However, because the primary endpoint of this study was cup placement accuracy, we believe that the sample size was appropriately calculated.

## Conclusions

We investigated the accuracy of acetabular cup placement in THA using a portable navigation system with a flip technique. The accuracy of cup alignment with the NAVBIT portable navigation system using the flip technique was significantly higher than that with the cup goniometer in the supine and lateral decubitus positions. This portable navigation system is useful because it is less susceptible to surgical positioning.
